# Unusual Contents of the Femoral Hernia

**DOI:** 10.5402/2011/717924

**Published:** 2010-10-17

**Authors:** Ahmed Alzaraa

**Affiliations:** Department of General Surgery, Leicester General Hospital, Leicester LE54PW, UK

## Abstract

Different contents in the femoral hernia have been reported in the literature, but herniation of the fallopian tube in a femoral hernia is very rare due to its normal anatomical position. *Case Presentation*. A female patient was admitted to the surgical ward for a lump in the right groin. Clinical examination confirmed a right femoral hernia. The patient underwent surgery to repair the hernia. Intraoperatively, the right uterine tube was found in the hernia. The tube was reduced back into the pelvic cavity and the hernia was repaired. After making good recovery, the patient was referred to the gynaecologist for further assessment. *Conclusion*. This case is educational as it highlights the importance of managing women with femoral masses with care.

## 1. Background

Herniation of the uterine tube is rare due to its anatomical position. If it happens, its status should be properly checked as the treatment depends on its viability.

## 2. Case Presentation

**Figure 1:**
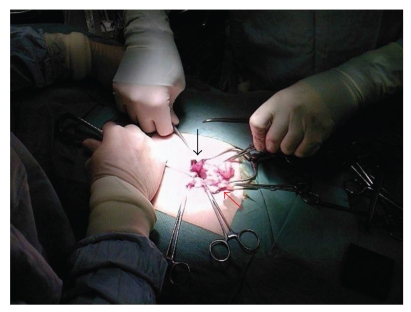
Fallopian tube (black arrow) in the explored femoral hernia (red arrow).

A 39-years-old patient was admitted to the surgical ward with a painful lump in the right groin which had been present for about three years. The lump had recently increased in size. She was investigated by the gynaecologists for primary infertility as she had failed to conceive for many years, but no cause was found. Clinical examination revealed a 5 cm × 5 cm tender, irreducible, nonpulsatile mass in the right groin just under the inguinal ligament. There was no cough impulse. A diagnosis of a femoral hernia was made. Exploration of the mass confirmed the diagnosis of the femoral hernia, but sac contained the right fallopian tube (Figure 1). The uterine tube was easily reduced back into the pelvic cavity as it looked healthy and the femoral hernia was repaired. The patient made good recovery, and she has been referred back to the gynaecology clinic for followup.

## 3. Discussion

The diagnosis of a femoral hernia is usually clinical. Most patients present as an emergency with symptoms and signs of intestinal obstruction. A typical femoral hernia presents as a tender, nonreducible swelling with no cough impulse and is situated below and lateral to the pubic tubercle. On exploration, it often contains only omentum, or may contain a knuckle of bowel known as Richter's hernia. The differential diagnoses include inguinal hernia, lipoma, saphena varix, enlarged lymph nodes, femoral artery aneurysm, sarcoma, obturator hernia, psoas abscess, psoas bursa, and in males, ectopic testis. 

Inguinal herniation of the adnexa is rare and is generally found in children with associated congenital abnormalities of the genital tract. In his analysis of 1,950 patients with inguinal hernias, Gurer et al. [1] found that most of his patients were adults with no genital abnormalities. The incidence of inguinal hernias containing ovary and fallopian tube in his study was 2.9%. Different contents in femoral hernias have been reported in the literature, such as appendix, small intestine, omentum, bladder, Meckel's diverticulum, ectopic testis, and stomach [2]. Herniation of the fallopian tube in a femoral hernia however is very rare due to its normal anatomical position as it lies at a lower level than the femoral ring [3]. In infants, the fallopian tube is near the internal inguinal ring and can more readily pass through this structure than later in life [4], due to the closure of the canal of Nuck. Due to its deep pelvic position, it is very difficult for the uterine tube to herniate in adults unless there is a sudden anomalous movement when the body is forcibly inclined towards the inguinal region, for example, or a pathological condition of the internal genital organs such as a uterine fibroid [4]. 

Patients with herniation of the fallopian tube might present with unusual symptoms which vary from intermittent attacks of abdominal pains, irreducibility and strangulation, increased hernia size during muscular effort, to those which resemble symptoms of ileus. Sometimes, a cord-like body in the sac leading to the hernial opening might be palpated. A vaginal bimanual examination help diagnose such cases by findings a deviation of the uterus towards the side of the hernia; for instance, the uterus being drawn over and inclined towards the side on which the hernia was situated, or the uterus is in a position of marked anteflexion and seemingly fixed [4].

If the tube is healthy on exploring the hernial sac, it should be reduced into the pelvic cavity. Otherwise, it should be removed if its viability is in doubt, gangrenous, or profound pathological changes have occurred [4]. In children, the peritoneal attachment of the fallopian tube and the ovary to the posterior wall has to be divided before reducing them if they are situated in the canal of Nuck as they are more or less fixed at this position [5].

## 4. Conclusion

We present a rare case of a femoral hernia containing a fallopian tube which clinicians should be aware of when managing women with femoral masses.

##  Conflict of Interests

The authors declare that they have no competing interests.

## Consent

Informed written consent was obtained from patient's relatives for publication of the manuscript and figures.
